# Fibers and Polyphenols in Diverticular Disease: From Pathophysiology to Management

**DOI:** 10.1111/nmo.70171

**Published:** 2025-09-26

**Authors:** Claudia Marinaccio, Annamaria Altomare, Benedetto Neri, Laura Restaneo, Dario Biasutto, Simone Carotti, Michele Cicala, Chiara Fanali, Michele Pier Luca Guarino

**Affiliations:** ^1^ Research Unit of Gastroenterology, Department of Medicine and Surgery Università Campus Bio‐Medico di Roma Roma Italy; ^2^ Department of Sciences and Technologies for Sustainable Development and One Health Università Campus Bio‐Medico di Roma Roma Italy; ^3^ Therapeutic GI Endoscopy Unit Fondazione Policlinico Universitario Campus Bio‐Medico Roma Italy; ^4^ Microscopic and Ultrastructural Anatomy Research Unit, Department of Medicine and Surgery Università Campus Bio‐Medico di Roma Rome Italy; ^5^ Operative Research Unit of Gastroenterology Fondazione Policlinico Universitario Campus Bio‐Medico Roma Italy; ^6^ Research Unit of Food Science and Human Nutrition, Department of Sciences and Technologies for Sustainable Development and One Health Università Campus Bio‐Medico di Roma Roma Italy

**Keywords:** dietary fiber, diverticular disease, gut microbiota, intestinal inflammation, nutritional therapy, polyphenols

## Abstract

**Backgrounds:**

Diverticular disease, particularly symptomatic uncomplicated diverticular disease (SUDD), significantly impacts patient quality of life and is increasing in prevalence, especially in Western countries. While its pathophysiology is multifactorial, diet—specifically low fiber intake—has been implicated as a key modifiable factor in disease development and progression. Fibers influence colonic motility and stool composition, potentially reducing the formation of diverticula and symptom severity. Polyphenols, bioactive compounds with antioxidant and anti‐inflammatory properties, may further protect intestinal integrity and modulate gut microbiota.

**Purpose:**

This narrative review explores emerging evidence on the role of dietary fiber and polyphenols in SUDD management. Despite promising mechanistic insights, current studies are limited by heterogeneity and methodological constraints. Personalized nutritional strategies focusing on fiber and polyphenol‐rich foods warrant further investigation to optimize therapeutic outcomes in SUDD.

## Graphical abstract

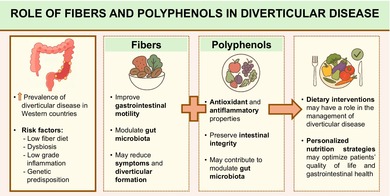

**Summary**

- Eating more fiber may help reduce symptoms of diverticular disease and improve gut function.
- Polyphenols, natural compounds found in plant‐based foods, could help protect the gut and reduce inflammation.
- A diet rich in fiber and polyphenols might become an important tool in managing diverticular disease without medication.

## 1 Introduction

Diverticulosis is a common gastrointestinal condition defined by the presence of diverticula, small mucosal pouches that protrude from the colonic wall by sliding into the *loci minorae resistentiae* [1]. While largely asymptomatic, approximately 25% of patients develop symptomatic uncomplicated diverticular disease (SUDD), characterized by recurrent abdominal pain, bloating, and altered bowel habits [2]. Overall, SUDD has a relevant negative impact on patients' quality of life [2, 3].

Epidemiological studies reported an increasing incidence of diverticular disease, particularly in Western countries [1]. The role of diet in diverticula formation has been widely suggested, as supported by the observation that the typical Western diet is characterized by low fiber content [1, 4]. The pathophysiology of SUDD is only partially defined, and apart from diet, genetic predisposition, low‐grade inflammation, and alterations in the gut microbiota have all been reported to be involved [5].

In this context, the role of dietary fiber remains a critical area of research, with evidence suggesting that increased fiber intake may reduce gastrointestinal symptoms and progression to diverticulitis [6, 7]. The present narrative review, therefore, aims to update the current evidence of the role of diet in the development and management of patients with SUDD with a focus on dietary fiber and emerging evidence about polyphenols.

## 2 Methods

The Pubmed and Scopus databases were consulted using the following search terms: “diverticulosis,” “diverticula,” “symptomatic uncomplicated diverticular disease” “diverticulitis” alone or in combination with “diet,” “fiber”, and “polyphenols.” The search was focused on full‐text papers published in English with no date restrictions.

## 3 Epidemiology

Diverticulosis is usually an incidental and asymptomatic condition observed in patients undergoing endoscopic or radiological examinations for other medical reasons [5]. Due to the incidental nature of the diagnosis, it is difficult to accurately estimate its incidence and prevalence, which is influenced by age and geographic location of the population [2, 5]. Indeed, it is widely reported that its prevalence increases with age, being as low as 10% in individuals < 40 years of age and up to 70% in those older than 80 years [1, 5, 8].

Gender differences have also been observed [9]. Diverticulosis is more prevalent in males than females gender until the 6th decade [9, 10]. A recent study reported that diverticulosis has a lower prevalence in premenopausal age women compared to postmenopausal age women, who show the same risk of developing diverticula as same‐age men, hinting at a possible key role for hormones in the development of diverticula [10].

In early epidemiological studies, diverticulosis has been described as a condition of “westernized” populations, with the highest incidence being registered in the United States, Western Europe, and Australia [1, 11]. This disparity has been attributed to differences in lifestyle and dietary habits, particularly the high‐fiber diet in the East versus the low‐fiber diet typical of Western countries [12]. Indeed, a higher consumption of dietary fibers has been associated with a decreased risk of diverticulitis in both male and female patients [13, 14]. A prospective study conducted in Sweden reported that immigrants from non‐Westernized countries carried a lower risk for hospitalization due to acute diverticulitis than natives [4]. The risk has been reported to progressively increase over time after settlement, suggesting the adoption of a western lifestyle as a potential risk factor for the development of diverticula [4].

Moreover, gender‐specific dietary habits may contribute to variations in fiber and polyphenol intake. Several population‐based studies have shown that women tend to consume more fruits and vegetables than men, both in terms of frequency and quantity. A recent Indian cohort study involving 800 adults demonstrated that women had a significantly higher daily fruit intake compared to men (*p* = 0.009), with over 55% of female participants consuming fruits daily versus 44% of males. Vegetable intake also showed a similar, although less pronounced, pattern of higher consumption among women [15]. Likewise, polyphenol‐rich food sources such as fruits and vegetables are more frequently consumed by women in various European populations. For instance, a review analyzing antioxidant intake patterns across multiple studies reported a consistent trend toward higher polyphenol intake in females, partly attributable to increased fruit and vegetable consumption [16]. These gender‐related differences in food preferences may influence not only fiber and polyphenol intake but also downstream effects on gut microbiota composition, intestinal function, and metabolic outcomes.

In the past decades, the prevalence of diverticulosis also increased in Asian populations [15, 16, 17]. A large‐scale study conducted in Japan showed that the prevalence of colonic diverticulosis has been rapidly increasing, from 1.6% to about 25% in 40 years [17, 18]. This trend is in accordance with the changes in mean dietary fiber intake observed in Japan, which showed a steady decrease in rice consumption (up to 35% reduction since the late 1970s) associated with an increase in that of animal‐derived food [19].

Variations in the anatomical distribution of diverticula depending on the geographic location of the population have also been noted [2, 5, 10]. In western industrialized nations, diverticula are more frequently located in the sigmoid colon [1]. In contrast, in Asian populations, diverticula are primarily located in the right side of the colon [20, 21]. Notably, right‐sided diverticula in these populations do not exhibit an age‐related increase, suggesting they may be an acquired condition [22].

Diverticular disease is usually defined as symptomatic diverticulosis ranging from a condition of SUDD to diverticulitis [23]. It is estimated that approximately 25% of individuals with diverticulosis develop SUDD while the progression to diverticulitis seems to occur in a minority of patients [2, 8]. Among these patients, only 12% of patients will progress to complications such as perforation, abscess, or fistula [5]. Although diverticula are more frequent in elderly individuals, diverticular disease seems to show a different age‐related trend with a higher rate of related hospitalizations and a more aggressive course in younger patients (< 45 years) [21, 22, 23]. Female patients have also been reported to have a higher hospitalization rate compared to male patients at all ages [24, 25].

## 4 Pathophysiology of Diverticular Disease

The path that leads from the development of diverticula to diverticular disease is still undefined, even though several hypotheses have been proposed. Recent evidence suggests that the different clinical phenotypes of this condition may be considered as a progression of the same pathological process [26]. While neuromuscular and connective tissue alterations seem to predispose to diverticula formation, other factors, such as low‐grade inflammation and alterations in gut microbiota, appear to be involved in the progression to symptomatic disease and diverticulitis [27, 28, 29].

### 4.1 Genetic Factors

The reported higher prevalence in Western countries compared to Asian populations of colonic diverticulosis, with a different diverticula distribution, suggests a key role of genetic susceptibility [27]. This is further supported by the early onset of diverticulosis in individuals with inherited connective tissue disorders, such as Marfan, Ehlers‐Danlos, and Williams‐Beuren syndromes [5, 26, 27]. Genetic associations have been reported with COL3A1 (type III collagen gene) [28], Reprimo (tumor suppressor gene) [29], polymorphisms in LAMB4 (laminin β 4 gene) [30], and TNFSF15 (T‐cell receptor gene) [31]. Two recent large genome‐wide association studies also identified multiple risk loci related to immunity, neuromuscular and epithelial function, as well as connective tissue integrity [32, 33, 34, 35].

### 4.2 Diet and Dietary Fibers

Since Burkitt and Painter [1] suggested a role for high dietary fiber intake as a protective factor for diverticular disease in 1971, multiple epidemiological studies have reported a potential protective effect of dietary fiber against the development of diverticulosis and diverticulitis [6, 36, 37, 38]. A review from Aldoori et al. [37] suggested that a fiber‐rich diet, mainly from the insoluble component of fruits and vegetables, and low in total fat and red meat, is associated with a decreased risk of diverticular disease. Indeed, a British prospective study reported that patients who followed a high‐fiber diet (≥ 25 g/day) had a 42% lower risk of hospitalization and mortality related to diverticular disease compared to participants who consumed < 14 g/day of fiber [38]. Overall, a recent systematic review and meta‐analysis of prospective cohort studies highlighted that consuming 30 g of fiber per day may entail a 41% risk reduction of developing diverticular disease [6]. Conversely, red meat and smoking habits were reported as possible risk factors for this condition [39, 40].

Low fiber intake, along with obesity and physical inactivity, plays a crucial role in shaping the diversity and function of the intestinal microbiota [36]. Fibers undergo fermentation by gut bacteria, leading to the production of short‐chain fatty acids (SCFAs), which are essential for enhancing the mucosal barrier and optimizing the immune function [41].

### 4.3 Dysbiosis

Currently, there is insufficient evidence to establish a correlation between dysbiosis and the pathogenesis of diverticular disease. However, preliminary data reported an increase of pro‐inflammatory bacteria in patients with symptomatic diverticular disease [42], while in asymptomatic diverticulosis, no difference was observed in microbiota composition when compared with healthy controls [43]. Alterations in intestinal microbiota composition, in association with inflammation and local trauma caused by faecalith, seem to be related to the progression to diverticulitis [44]. *Firmicutes* and *Bacteroidetes* represent more than 90% of the phyla composing gut microbiota, contributing to the maintenance of gut homeostasis through their metabolic and immunomodulatory functions, as well as by preserving the structural integrity of the mucosal barrier of the gut [45]. The *Firmicutes/Bacteroidetes* ratio has also been proposed as a biomarker of a healthy gut [46]. As reported for other gastrointestinal conditions, such as irritable bowel syndrome (IBS) and inflammatory bowel diseases, an increase in the *Firmicutes/Bacteroidetes* ratio in diverticular disease has also been reported [47].

In 2017, Barbara et al. [48] reported a decreased abundance of *Clostridium* cluster IX, *Fusobacterium*, and *Lactobacillaceae* in patients with SUDD compared to patients with diverticulosis. These bacteria exert an anti‐inflammatory action at the mucosal level, respectively producing propionate, butyric, and lactic acid [49]. In the same study, an inverse correlation between macrophages in the peridiverticular area and the presence of *Akkermansia muciniphila* , whose depletion has been associated with gut inflammation, has also been reported [48]. *Akkermansia muciniphila* is a Gram‐negative bacterium that produces propionate in the presence of vitamin B12. Beyond its role in short‐chain fatty acid production, this bacterium appears to exert anti‐inflammatory and immunomodulatory effects, potentially linked to its mucin‐producing capacity [50]. Bloating, which represents a common symptom of this condition, has also been linked to gut microbiota alteration. A pilot study conducted in 2017 found that an increase in *Ruminococcus*, along with a decrease of *Roseburia*, was associated with a more severe bloating [51]. This correlation may be related to the ability of *Ruminococcus* to release hydrogen gas through the fermentation of carbohydrates, leading to the increased bloating referred to by these patients [51]. The authors also observed that microbial composition was significantly correlated with symptom severity scores, reinforcing the hypothesis that dysbiosis may play an active role not only in inflammation but also in functional symptom generation [51].

However, it has to be pointed out that the currently available evidence is burdened by a few methodological limitations that complicate data interpretation. Indeed, some of the reported studies did not account for the use of antibiotics before the collection of the sample [42, 47]. Moreover, differences in sample type and analytical techniques among the available studies are partly due to the progressive adoption of new technologies over time [47]. These data support the need for further investigation into the metabolome to propose microbiota‐targeted therapies as a potential tool to manage gastrointestinal symptoms in diverticular disease [44, 45, 47].

### 4.4 Low‐Grade Inflammation

Emerging evidence suggests that chronic low‐grade inflammation may contribute to symptom development in diverticular disease, although its role in diverticula formation remains under investigation [5, 52]. Recent studies reported a significant increase in IL‐10, TNF‐alpha, and fecal calprotectin in patients with SUDD compared with asymptomatic subjects [53, 54, 55].

A role for oxidative stress in the pathogenesis of diverticular disease has also been suggested [56, 57, 58]. Indeed, patients with diverticular disease showed higher levels of oxidative stress biomarkers (sNox2‐dp, H^2^O^2^, and isoprostane) when compared to healthy subjects, associated with a decrease in systemic antioxidant capacity [57]. Gut microbiota may also be involved in oxidative stress due to an increase in gut permeability, increasing the possibility of bacteria translocation in the diverticular area [59]. Alterations in gut microbiota composition may compromise intestinal barrier function, leading to increased permeability and facilitating bacterial translocation, which in turn can exacerbate local inflammation and oxidative stress [57]. According to this hypothesis, lipopolysaccharide (LPS) binding to *TLR4* may cause a cell shift in colonic smooth muscle cells from a contractile to a proliferative phenotype [59].

The binding of LPS to Toll‐like receptor 4 (TLR4) on colonic smooth muscle cells can induce a phenotypic switch from a contractile to a proliferative state, contributing to muscular alterations observed in diverticular disease [59]. An important stress‐related damage has also been reported in the mitochondria of patients with diverticular disease [58]. Overall, even though available evidence is not definitive, the reported studies prompted further research regarding potential therapeutic strategies for prevention of symptomatic diverticular disease, including the use of antioxidants [56].

## 5 Clinical Characteristics of Diverticular Disease

Currently, a standard definition and classification of diverticular disease is lacking. Indeed, different terminologies have been proposed [60, 61]. According to the most recent guidelines for the management of diverticular disease of the colon, it is possible to differentiate diverticulosis, which is usually an incidental finding and refers to the presence of diverticula without symptoms and signs of inflammation, from diverticular disease, which encompasses different stages of this condition [23]. Diverticular disease encompasses SUDD, diverticulitis, further divided into acute and chronic, uncomplicated and complicated, and diverticular bleeding [62].

In accordance with the 2024 Italian guidelines, a possible diagnosis of SUDD is suggested in the presence of abdominal pain referred to the left lower quadrant associated with changes in bowel habits or bloating, with no evidence of inflammation [23]. The clinical features of SUDD often overlap with IBS characteristics, thus complicating the differential diagnosis [63]. However, the differences in terms of gender distribution, age at onset, and duration of symptoms may help to identify these two conditions. Indeed, SUDD is generally more frequent in older male patients, while pain is more often localized in the left lower quadrant, usually prolonged (> 24 h) with long remission periods [64]. Differently, IBS is usually reported in young female patients with diffuse and recurrent abdominal pain lasting < 24 h [63]. Furthermore, since fecal calprotectin seems to be increased in SUDD, it has been proposed as a biomarker to differentiate SUDD from IBS [23, 64]. However, due to a lack of evidence, the role of fecal calprotectin in SUDD diagnosis is still undefined [55]. As reported in a cohort study conducted by Comparato et al. [3], SUDD seems to impair both mental and physical quality of life. This finding may account for the current effort in researching new therapeutic strategies to prevent symptomatic uncomplicated disease.

The pathophysiological process that leads from asymptomatic diverticulosis to acute diverticulitis seems to be related to the obstruction of diverticula by fecaliths, causing mucosal injury, local ischemia, and longer exposure to intraluminal pathogens due to bacterial translocation [44]. Patients with acute diverticulitis usually present with left‐sided abdominal pain and tenderness, potentially associated with fever and/or changes in bowel habits, rectal discharge of mucus or bleeding [23].

Patients with acute colonic diverticulitis can be divided into those with uncomplicated and those with complicated diverticulitis, depending on the presence of abscess, fistula, peritonitis/perforation, or diverticular bleeding [5]. As reported in a 2016 population‐based study, peri‐colonic abscess and peritonitis appear to be the most frequent complications, respectively 69% and 27%, while fistula and obstruction seem to be less frequent [65]. Of note, diverticular hemorrhage, which occurs in 5%–15% of patients with diverticulosis, represents a frequent complication that in one third of patients can be massive and require hospitalization [22]. Diverticular bleeding is caused by the rupture of vasa recta and is usually characterized by the absence of pain and no sign of inflammation around the area involved by the bleeding [66]. This histological finding seems to dissociate the inflammatory process from the bleeding onset [66]. Conversely, cardiovascular disease (i.e., arterial hypertension and ischemic heart disease), diabetes mellitus, and the use of anticoagulant drugs seem to be associated with a higher risk of diverticular hemorrhage [66].

When presenting with signs of sepsis, formation of a mass or peritonitis at abdominal examination, signs of intestinal obstruction such as vomiting or no passage of stool and gas, indirect signs of fistula (i.e., fecaluria, pneumaturia, or fecal discharge from the vagina), patients should be directed for urgent hospital evaluation to confirm the diagnosis of diverticulitis and assess any potential complication [23]. Since clinical evaluation and laboratory tests are not specific for diverticulitis, contrast‐enhanced computed tomography (CE‐CT) should be performed, directing the choice of treatment according to the severity of diverticulitis [67, 68]. The radiological criteria for diverticulitis are inflamed diverticula, thickening of the intestinal wall to over 3 mm, and increased contrast enhancement [23, 69]. In 2005, Kaiser et al. [70] revised the well‐known Hinchey classification, which was originally based on intraoperative findings. The modified Hinchey classification relies on CT findings and is currently one of the most commonly used systems to assess the severity degree of acute diverticulitis (from mild to generalized purulent peritonitis) [68]. This classification first introduced the concept of mild clinical diverticulitis (Stage 0) based on clinical and laboratory findings (i.e., pain, elevated white blood cell count, and fever) without any supporting surgical or imaging findings [70]. The following stages describe pericolic inflammation or phlegmon (Stage Ia), pericolic abscess (Stage Ib), pelvic/intra‐abdominal or retroperitoneal abscess (Stage II), generalized purulent peritonitis (Stage III), and fecal peritonitis with an open perforation of the bowel (Stage IV) [70]. In broad terms, mild diverticulitis (Stage 0–Ia) can be managed with medical treatment (broad‐spectrum antibiotics), moderate severity Stages (Ib–II) may respond to conservative treatment, or less frequently, need a percutaneous or surgical approach, while more severe cases (Stage III–IV) always require surgery [62].

According to a recent meta‐analysis, the recurrence rate after a first episode of acute uncomplicated diverticulitis is 12.9%, with a significant difference between European (14.7%) and Asian countries (9.2%) [71]. Among the risk factors promoting diverticulitis recurrence, Buchs et al. [72] also found that patients with a CRP concentration higher than 240 mg/L had a 6‐month recurrence risk of 22% compared with the 8.2% of those with lower levels. Concerning acute complicated diverticulitis in the presence of an abscess treated with a percutaneous approach, studies report a recurrence rate between 28% and 50% [73]. This significant discrepancy is due to the different characteristics of the studies, such as the follow‐up duration or definition of recurrent diverticulitis, that may influence the result. As expected, the recurrence rate significantly drops (2.1%) in patients with severe diverticulitis who underwent colectomy [74].

## 6 Medical Treatment of SUDD

Diverticular disease is a heterogeneous condition that requires different therapeutic approaches (medical, radiological, surgical) according to the severity. Since diverticulosis is an asymptomatic condition, current European guidelines do not recommend any medical treatment [62].

As anticipated, despite having a high prevalence and a negative impact on life quality, the management of SUDD is still uncertain, and guideline recommendations are based on a low level of evidence due to the scarcity of current literature [75]. The role of dietary fiber for the improvement of gastrointestinal symptoms seems to be related to its contribution to gut microbiota composition and stool transit time [64]. Two recent meta‐analyses reported a potential symptom improvement with a high‐fiber diet or fiber supplementation. However, the studies included presented several limitations, including heterogeneity of diets, use of different fiber supplementation (*glucomannan, ispaghula, bran, plantago ovata*, and *methylcellulose*), and lack of a standardized treatment [76]. Therefore, the quality of evidence is low; thus, more studies are needed to include a high‐fiber diet as a strong recommendation in future guidelines and clinical practice [64]. Taking into account the alterations of gut microbiota reported in patients with SUDD, recent studies have suggested a protective role for probiotics by decreasing pathogenic bacteria and enhancing mucosal defence [48]. Most of these studies investigated the impact of *Lactobacilli* and *Bifidobacteria*; however, the differences among doses and association with other therapies (like mesalamine, balsalazide) limit the possibility of comparing their results [77].

Currently, guidelines also suggest the use of cyclic nonabsorbable antibiotics in association with a high‐fiber diet to reduce SUDD‐related symptoms [23]. Rifaximin is a non‐aminoglycoside semisynthetic nonsystemic antibiotic derived from rifamycin that inhibits bacterial RNA synthesis [78]. The rationale for using rifaximin in SUDD is based on its eubiotic effect on gut microbiota, reducing the low‐grade inflammation that characterizes this condition [78]. It has been suggested that rifaximin enhances the effects of a high‐fiber diet by limiting the overgrowth of gut microflora. This, in turn, may lead to a reduction in the production of bacterial gases such as hydrogen (H_2_) and methane (CH_4_) [79]. Accordingly, a 2011 meta‐analysis reported a higher frequency of symptom remission at 1 year in patients with SUDD treated with a high‐fiber diet associated with 7‐day monthly cycles of rifaximin when compared with controls treated with only a high‐fiber diet [80].

Mesalamine, an aminosalicylate with anti‐inflammatory properties, has also been proposed in the management of SUDD, though its recommendation is still controversial [23, 64]. While large‐scale trials such as PREVENT1 and PREVENT2 didn't report any significant improvement in patients' QoL using mesalamine, real‐life studies reported a clinical improvement in symptom relief in patients treated with this compound when compared to patients treated with a placebo [81]. To date, international guidelines do not recommend the use of mesalamine in patients with SUDD due to the inconsistency of available literature [23, 62].

## 7 Role of the Diet in the Management of DD

The therapeutic management of SUDD is still uncertain, as testified by the low‐level evidence of current guidelines recommendations, due to the limited availability of evidence [75]. A significant discrepancy regarding the incidence of diverticular disease between Western countries and rural Africa has been described since the seventies [1]. A potential inverse correlation between dietary fiber intake and the incidence of diverticular disease has been hypothesized, suggesting that a high‐fiber diet may play a protective role in diverticula development [1, 7, 13, 36].

### 7.1 Fibers

Fibers are commonly classified into soluble and insoluble types, which differ in fermentability and their effect on stool consistency and transit time [7, 82]. Fibers retain water in the intestinal lumen, increasing stool weight and reducing transit time [82]. By lowering the pressure exerted by stool in the weaker areas of the colonic lumen, adequate intake of fiber may reduce the likelihood of developing diverticula [1]. In addition, dietary fibers undergo fermentation by intestinal bacteria, leading to the production of SCFAs, which improve colonic mucosal integrity [6]. Based on these properties, in the last decades, several studies investigated the potential role of a high‐fiber diet and fiber supplementation in SUDD management and prevention of acute diverticulitis [76]. Two systematic reviews have examined the role of dietary fiber in the management of SUDD, suggesting a potential benefit in symptom improvement and stool regulation [76]. However, both highlighted significant methodological limitations, including heterogeneity in fiber types and dosages, inconsistencies in study design, and lack of standardized treatment protocols. These issues prevent strong conclusions and limit the current strength of evidence supporting fiber supplementation in clinical practice [7]. Aune et al. [6] reported a correlation between different types of fibers and diverticular disease and suggested a reduction in the risk of acute diverticulitis with 10 g/die of fruit fiber and cereal.

A large prospective cohort study found that male patients following a fiber‐rich, prudent diet had a lower risk of diverticulitis compared to those on a Western diet [83]. Accordingly, international guidelines recommend high fiber intake and lifestyle changes to help reduce symptoms and possibly prevent diverticulitis [23, 62, 67, 84].

Nevertheless, not all evidence consistently supports a protective role of dietary fiber in diverticular disease. A large cross‐sectional study by Peery et al. [85], involving over 2000 asymptomatic individuals undergoing screening colonoscopy, found that higher fiber intake was significantly associated with an increased prevalence of diverticulosis. This association was dose‐dependent and observed across all fiber types, including total, grain, soluble, and insoluble fiber. These findings directly challenge the long‐standing hypothesis that a high‐fiber diet prevents the formation of diverticula [85].

One possible explanation is the phenomenon of reverse causation. Individuals with a known family history or an early diagnosis of diverticulosis may increase their fiber intake as a result of medical advice or personal efforts to reduce future risk. In such cases, the observed association could reflect a consequence of the disease rather than a true causal relationship. It is also critical to emphasize that this study focused specifically on asymptomatic diverticulosis, which may involve distinct pathophysiological mechanisms compared to SUDD or diverticulitis, commonly addressed by dietary interventions. These discrepancies highlight the need for well‐designed prospective and mechanistic studies to define better the role of fibers in the pathogenesis of diverticular disease [85].

An additional and underestimated property that may be involved in the positive effect shown by fibers is the antioxidant capacity [86]. Antioxidant dietary fibers can be described as fibers with a significant antioxidative capacity, mainly coming from polyphenols. Indeed, an oxidative imbalance has been reported in patients with diverticular disease, showing higher levels of oxidative stress biomarkers and a decreased antioxidant capacity when compared to healthy subjects [57].

### 7.2 Polyphenols

**FIGURE 1:**
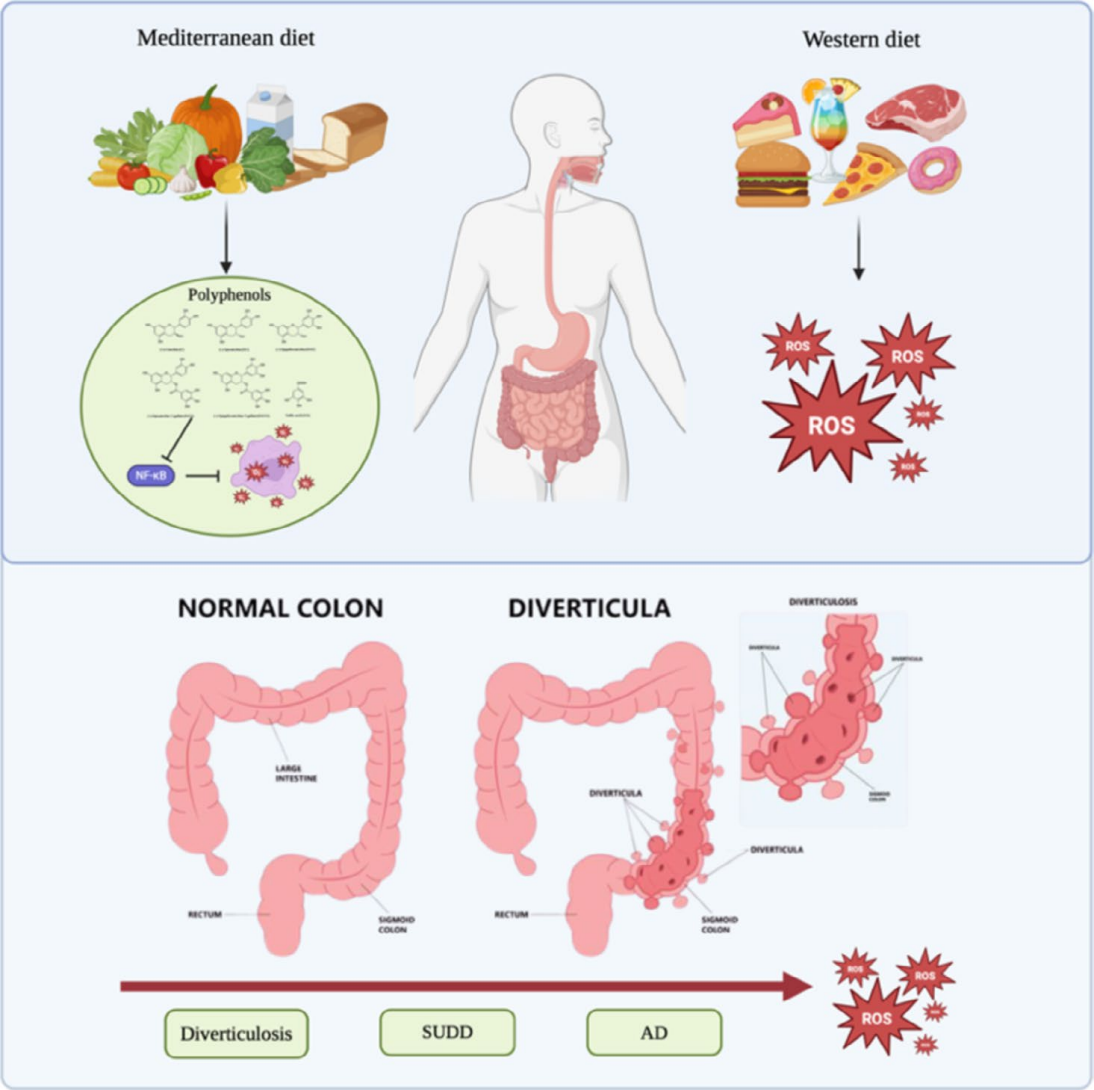
The illustration highlights the role of dietary patterns in modulating intestinal oxidative stress and the pathogenesis of diverticular disease. A Mediterranean diet, enriched in polyphenols, exerts anti‐inflammatory effects by inhibiting NF‐κB activation and mitigating ROS generation. Conversely, a Western diet promotes oxidative stress and inflammation through excessive ROS production. The progression from a healthy colon to diverticulosis, symptomatic uncomplicated diverticular disease (SUDD), and acute diverticulitis (AD) is depicted, illustrating a continuum characterized by increasing oxidative damage.

Polyphenols, a diverse group of bioactive compounds characterized by phenolic structures and strong electron‐donating properties, are well recognized for their potent antioxidant activity [87]. Their relevance becomes particularly evident when considering the pathophysiological continuum from asymptomatic diverticulosis to overt diverticulitis, a transition in which chronic low‐grade inflammation, oxidative stress, and epithelial barrier dysfunction are key contributors (Figure 1). Given the central role of oxidative stress and the excessive generation of ROS in the pathogenesis of diverticulitis, it is plausible that polyphenols exert protective effects by scavenging free radicals, thereby contributing to the preservation of intestinal epithelial integrity.

These considerations highlight the potential for a synergistic interaction between dietary fiber and polyphenolic compounds, warranting further investigation into their combined role in promoting gastrointestinal health [87, 88].

Moreover, polyphenols modulate key signaling pathways, such as NF‐κB, thereby attenuating the inflammatory response. They may also favorably influence gut microbiota composition, further enhancing intestinal barrier function. Collectively, these properties make polyphenols promising bioactive molecules for mitigating the oxidative and inflammatory processes underlying diverticulitis [86, 89].

Polyphenols can be classified into several main categories:

- Flavonoids, including flavanols (quercetin, kaempferol, myricetin), flavan‐3‐ols (catechins, epicatechins), and anthocyanins are present in apples, onions, tea, leafy greens, berries, and cocoa [90].
- Phenolic acids, such as chlorogenic, ferulic, and p‐coumaric acid are found in whole grains, nuts, and some vegetables [90].
- Stilbenes, such as resveratrol from grapes and red wine, offer anti‐inflammatory benefits [90].
- Lignans, present in whole grains, flaxseeds, and walnuts, contribute to antioxidant effects [90].

Polyphenols are ubiquitously present in plant‐based foods including fruits– notably apples, pears, and prunes [13, 91]—vegetables, whole grains, cocoa, walnuts, and flaxseeds, and are integral to mitigating oxidative stress and modulating inflammatory responses within the gastrointestinal tract, a factor of particular relevance in conditions such as diverticulitis [13, 86].

Although a universal threshold for the minimum or maximum polyphenol intake remains undefined and individual metabolic differences further influence their bioavailability, certain foods naturally provide substantial quantities of these compounds. Polyphenol content data were consistently retrieved from the Phenol‐Explorer database, where each food item is associated with a specific Food ID (Table 1). For instance, apples (Food ID 29) typically contain several 100 mg of polyphenols per 100 g, with variability according to cultivar and agricultural practices [91]. Consequently, a medium apple weighing approximately 150–200 mg can significantly contribute to daily polyphenol intake, even if it may not independently reach the proposed optimal range of 500–1000 mg for health benefits [92]. In contrast, pears (Food ID 51) provide around 60 mg of total polyphenols per 100 g, while prunes (Food ID 39) deliver approximately 160 mg per 100 g [91]. These quantitative differences, coupled with the high polyphenolic content of apples, underscore the nutritional rationale for incorporating a diverse array of these fruits into the diet to harness their cumulative antioxidant and anti‐inflammatory properties [13]. This combination fosters a synergistic effect on gut health: fiber enhances intestinal transit and acts as a prebiotic, while also prolonging polyphenol retention in the gut. This extended exposure facilitates microbial metabolism of polyphenols into bioactive metabolites with enhanced antioxidant and anti‐inflammatory properties [89, 90]. The polyphenol composition and content of these fruits are detailed in Table 1.

**TABLE 1:** Polyphenol composition and average content in selected fruits associated with diverticulitis risk reduction. This table summarizes the main classes of polyphenols and their average content (in mg per 100 g of fresh weight) in selected fruits that have shown protective associations with diverticular disease. Data are sourced from the Phenol‐Explorer database, and the listed foods include their respective database identifiers for traceability.

Food (Phenol‐Explorer ID)	Main polyphenol classes	Polyphenol content (mg/100 g fresh weight)
Apples (ID 29)	Flavanols (catechin, epicatechin), phenolic acids (chlorogenic acid), flavonols (quercetin)	Catechin (mg/100 g): 1.95–6.95; Epicatechin (mg/100 g): 7.76–22.56; Clorogenic acid (mg/100 g): 1.00–8.00
Pears (ID 51)	Flavanols, phenolic acids, flavonols	Catechin (mg/100 g): 0.50–1.50; Epicatechin (mg/100 g): 0.80–2.00; Clorogenic acid (mg/100 g): 0.90–3.00
Prunes (ID 39)	Phenolic acids, flavanols, anthocyanins	Clorogenic acid (mg/100 g): 5.00–10.00; Catechin (mg/100 g): 1.00–3.00; Epicatechin (mg/100 g): 0.50–1.50; Total anthocyanins (mg/100 g): 2.00–5.00
Tropical fruits	Flavonols (quercetin, kaempferol), phenolic acids	Quercetin (mg/100 g): 0.20–0.50; Kaempferol (mg/100 g): 0.10–0.30; Ferulic acid (mg/100 g): 0.50–1.00

In whole grains, the concurrent presence of fiber, lignans, and phenolic acids promotes the production of SCFAs via fiber fermentation and further modulates the microbiota, thereby mitigating inflammatory responses and preserving epithelial barrier integrity [13, 89].

### 7.3 Synergic Interplay Between Dietary Fibers and Polyphenol‐Rich Foods

Overall, the combined intake of polyphenol‐rich foods and dietary fiber represents a promising, multifactorial strategy for the prevention and management of diverticulitis. Fiber contributes to reducing intraluminal pressure and modulating colonic transit, while polyphenols exert protective effects by counteracting oxidative stress and chronic low‐grade inflammation. This synergistic interplay supports microbiota homeostasis and the preservation of the intestinal barrier, ultimately lowering the risk of disease recurrence [13].

Findings from a large perspective cohort study in women further reinforce this concept, showing that higher total fiber intake, particularly from fruit and cereal sources, was associated with a significantly reduced risk of incident diverticulitis [13]. Notably, the study identified whole fruits such as apples, pears, and prunes as particularly protective, highlighting the importance of food quality alongside quantity [88]. However, it is essential to recognize that the practical implementation of such dietary recommendations can be challenging. Individuals with a history of diverticular disease often develop restrictive eating patterns, frequently avoiding polyphenol‐rich and FODMAP‐containing foods out of fear of symptom recurrence. This behavior may stem from hospital‐imposed dietary limitations during acute episodes—such as clear‐fluid or low‐residue diets—which can create lasting perceptions of “unsafe” foods. Qualitative studies describe this as a “fear of food” cycle, where avoidance behaviors persist even in remission and lack strong evidence of benefit [93]. Moreover, this habit may lead to the exclusion of beneficial compounds and a reduction in overall dietary diversity. In this context, nutritional counseling emerges as a fundamental tool to help patients identify well‐tolerated food sources and to gradually reintroduce polyphenol‐ and fiber‐rich foods in a personalized and symptom‐conscious manner.

Furthermore, although fiber intake was traditionally restricted in the clinical management of diverticular disease, current evidence [13] supports its protective role. Personalized nutrition strategies guided by qualified professionals are, therefore, crucial not only for correcting outdated misconceptions but also for promoting long‐term dietary adherence, patient empowerment, and improved intestinal health.

## 8 Conclusions

Historically, dietary interventions have often been discouraged in the management of SUDD due to concerns that certain foods might exacerbate symptoms or lead to complications. However, current guidelines have changed this perspective, recognizing the pivotal role of nutrition in both symptom management and disease prevention. Dietary fibers are now acknowledged for their benefits in enhancing gastrointestinal motility, modulating the gut microbiota, and reducing intraluminal pressure. In particular, polyphenols, found abundantly in plant‐based foods, exhibit antioxidant and anti‐inflammatory properties that may further improve gastrointestinal health. Emerging evidence suggests that polyphenols can modulate gut microbiota composition and function, contributing to the maintenance of intestinal barrier integrity and to the reduction of oxidative stress. Given the interindividual variability in responses to dietary components, personalized nutrition strategies are essential. Not only the quantity, but also the quality and individual tolerability of foods must be considered. Therefore, a proper balance in the intake of foods that contain both FODMAPs and polyphenols by adjusting their introduction based on the patients' clinical response seems to be highly useful. Tailoring dietary recommendations to individual tolerances and metabolic profiles ensures optimal intake of fibers and polyphenols, thereby enhancing therapeutic outcomes and promoting long‐term adherence to dietary modifications.

## Author Contributions

Conceptualization: C.M., A.A., B.N., and M.P.L.G. Methodology: C.M., B.N., A.A., and M.P.L.G. Data curation: C.M., L.R., B.N., D.B. Writing – original draft preparation: C.M., L.R., A.A., B.N. Writing – review and editing: A.A., S.C., C.F., M.C., B.N., and M.P.L.G. Supervision: C.F., M.C., S.C., and M.P.L.G. all authors have read and agreed to the published version of the manuscript.

## Conflicts of Interest

The authors declare no conflicts of interest.

## Data Availability

The data that support the findings of this study are available from the corresponding author upon reasonable request.
